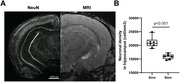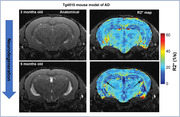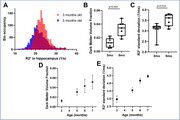# Novel MRI method enables in vivo quantification of pre‐atrophic neurodegeneration in a mouse model of Alzheimer’s Disease

**DOI:** 10.1002/alz.087522

**Published:** 2025-01-09

**Authors:** Michal R Tomaszewski, Alexander L Sukstansky, Hyking D Haley, Xiangjun Meng, Corin O Miller, Dmitriy A. Yablonskiy

**Affiliations:** ^1^ Merck & Co., Inc., West Point, PA USA; ^2^ Washington University, St Louis, MO USA; ^3^ Merck & Co, Inc, West Point, PA USA; ^4^ Washington University, St. Louis, MO USA

## Abstract

**Background:**

Robust methods are needed for preclinical evaluation of novel Alzheimer Disease (AD) therapies to accelerate drug discovery. Quantitative Gradient Recalled Echo (qGRE) MRI shows significant promise to provide insight into neurodegeneration in AD prior to atrophy development in humans, with qGRE R2t* metric (tissue‐specific subcomponent of R2*) highlighting areas of low neuronal density (doi:10.3233/JAD‐210503). In this study a novel qGRE method is shown to non‐invasively measure the longitudinal neuronal loss in the hippocampus of a mouse model of AD tauopathy Tg4510.

**Method:**

Tg4510 (n=15) and wild type (WT, n=7) mice underwent MRI (7T field strength) at 3, 5, 6, 7 months old (WT at 3 and 5mo only). Mice were isoflurane‐anesthetized to minimize BOLD contribution to R2* signal. 3D Multi‐GRE sequence was used to generate R2* maps. Light‐sheet microscopy of the brains stained with NeuN and MBP was used to visualize neuronal nuclei and myelin content respectively.

**Result:**

Significant decrease in NeuN staining between 3mo and 6mo was observed in the hippocampus of Tg4510, indicating a decrease of over 30% in neuronal density (p<0.001, Figure 1), validating the mouse model. Longitudinal analysis showed clear changes in R2* values in the Tg4510 hippocampus undergoing neurodegeneration between 3 and 5 months old (Figure 2). Histogram analysis revealed patterns of increase in low R2* value (Dark Matter, DM), and broadening of R2* distribution. These were quantified as significant increase in both DM Volume Fraction (DMVF) and R2* Standard Deviation (SD) in Tg4510 mice (Figure 3, p=0.004/p=0.016 DMVF/SD) but not in WT controls (p>0.25). Further monotonical increase was also observed in both metrics in time. A significant negative correlation was observed between myelin content and the DMVF (p=0.01, r=‐0.76) in the hippocampus, suggesting sensitivity of the technique to the loss of myelinated axons.

**Conclusion:**

In this study a method is proposed for using R2* relaxometry as a direct biomarker of the neuronal loss. Low R2* volume fraction (DM) and the heterogeneity of R2* both show high sensitivity for capturing longitudinal neurodegeneration. The presented technique, together with accompanying histological validation can be readily applied in preclinical models of neurodegeneration for pharmacodynamics and mechanism of action assessment.